# Corrugated Photoactive Thin Films for Flexible Strain Sensor

**DOI:** 10.3390/ma11101970

**Published:** 2018-10-13

**Authors:** Donghyeon Ryu, Alfred Mongare

**Affiliations:** Department of Mechanical Engineering, New Mexico Tech; Socorro, 87801, NM, USA; alfred.mongare@student.nmt.edu

**Keywords:** P3HT, PEDOT:PSS, flexible sensor, strain sensor, photoactive self-sensing thin films

## Abstract

In this study, a flexible strain sensor is devised using corrugated bilayer thin films consisting of poly(3-hexylthiophene) (P3HT) and poly(3,4-ethylenedioxythiophene)-polystyrene(sulfonate) (PEDOT:PSS). In previous studies, the P3HT-based photoactive non-corrugated thin film was shown to generate direct current (DC) under broadband light, and the generated DC voltage varied with applied tensile strain. Yet, the mechanical resiliency and strain sensing range of the P3HT-based thin film strain sensor were limited due to brittle non-corrugated thin film constituents. To address this issue, it is aimed to design a mechanically resilient strain sensor using corrugated thin film constituents. Buckling is induced to form corrugation in the thin films by applying pre-strain to the substrate, where the thin films are deposited, and releasing the pre-strain afterwards. It is known that corrugated thin film constituents exhibit different optical and electronic properties from non-corrugated ones. Therefore, to design the flexible strain sensor, it was studied to understand how the applied pre-strain and thickness of the PEDOT:PSS conductive thin film affects the optical and electrical properties. In addition, strain effect was investigated on the optical and electrical properties of the corrugated thin film constituents. Finally, flexible strain sensors are fabricated by following the design guideline, which is suggested from the studies on the corrugated thin film constituents, and the DC voltage strain sensing capability of the flexible strain sensors was validated. As a result, the flexible strain sensor exhibited a tensile strain sensing range up to 5% at a frequency up to 15 Hz with a maximum gauge factor ~7.

## 1. Introduction

Flexible strain sensors are widely used in various applications (e.g., biomedical devices, flexible displays, soft robotics, automobiles, and aerospace structures, among many others). Unlike conventional strain sensors, the flexible strain sensors exhibit an extended range of strain measurements as well as mechanical resiliency. To accomplish the design goal of devising flexible strain sensors, researchers have proposed various approaches by designing materials and sensing composites [1,2,3,4,5], modifying physical configurations [6,7], and patterning brittle components [8,9]. While the state-of-the-arts have showcased impressive sensing capability and mechanical resiliency, a majority of the flexible sensors use piezoresistive materials. Yet, the piezoresistive flexible strain sensors have intrinsic limitations, such as energy dependency and a singular sensing mode, among some others.

To overcome the intrinsic limitations of the piezoresistive strain sensors, researchers suggested novel flexible strain sensor technologies [2,5,10,11,12,13,14,15,16,17,18,19]. Piezoelectric materials-based strain sensors showed decent features in strain sensing performance [13,14]. It was reported that piezoelectric (1−x)Pb(Mg_1/3_Nb_2/3_)O_3_−xPbTiO_3_ nanowire-based elastomeric composites generated electric voltage, which exhibited a linear relationship with applied tensile strain when exposed to tensile strain [14]. Mechanoluminescent (ML)-based elastomeric composites exhibited strain sensing capability based change in ML light intensity as well as color of light emission under tensile or compressive strains [15,16,17,18]. It was reported that ML copper-doped zinc sulfide (ZnS:Cu) micro-particles, which are embedded in the elastomeric matrix, generated ML light under tension, of which luminescence showed a linear relation with applied tensile strain up to ~35%, under tensile loading and unloading cycles [17]. The new classes of flexible strain sensing composites present promising prospects with multifunctional capabilities of energy conversion from mechanical energy to electrical or radiant energy.

Of many multifunctional composites employed for devising self-powered strain sensors, multifunctional mechano-luminescence-optoelectronic (MLO) composites were recently proposed to sense tensile strain using direct current (DC) [11,20]. DC (i.e., electrical energy) was generated from the MLO composites subjected to cyclic tensile loading and unloading (i.e., mechanical energy). The MLO composites consist of two functional constituents, such as mechano-optoelectronic (MO) poly(3-hexthylthiophene) (P3HT)-based self-sensing thin film and ML ZnS:Cu-embedded elastomeric composites. On one hand, it was shown that the MO P3HT-based thin film generated DC under light, which varied with applied tensile strain [21,22,23]. The strain sensor fabricated with MO P3HT-based thin film did not require external electrical energy with a gauge factor of 2 or higher when a carbon nanotube is doped into the P3HT-based thin films. On the other hand, ML ZnS:Cu-based elastomeric composites, which are another functional constituent of MLO composites, emit light in response to mechanical stimuli [15]. Accordingly, the MLO composites were designed to measure strain using DC voltage output as a sensor signal via mechanical-radiant-electrical energy conversion. Nevertheless, the MLO composites-based strain sensor did not show a large range of tensile strain sensing mainly due to cracks occurring in brittle constituents. In particular, the top and bottom electrodes developed cracks at 1% strain or higher. It is critical to make flexible MO P3HT-based strain sensor for improving strain sensing range and mechanical resiliency of the MLO composites.

In this study, to enhance mechanical resiliency as well as the strain sensing range of the P3HT-based thin film strain sensor, a flexible strain sensor is designed by inducing buckling in the thin film constituents. Also, the brittle aluminum top electrode (i.e., cathode) is replaced with liquid electrode. As a functional constituent, poly(3,4-ethylenedioxythiophene)-poly(styrenesulfonate) (PEDOT:PSS) thin film as a bottom electrode (i.e., anode) is required to be electrically conductive and translucent under tensile strain. As another constituent, the MO P3HT-based self-sensing thin film is expected to exhibit radiant-electrical energy conversion under tensile strains while maintaining DC-based strain sensing capability.

Here, we suggest design guidelines for the corrugated PEDOT:PSS and P3HT-based thin film constituents for devising a flexible strain sensor. First, the corrugated PEDOT:PSS thin films, which are fabricated at various pre-strains and have a different number of layers, will be characterized to understand how sheet resistance and optical transmittance vary with design parameters (i.e., pre-strain and the number of layers). Besides, electrical and optical properties of the corrugated PEDOT:PSS thin films will be studied when various levels of tensile strains are applied onto the thin films. Second, the effect of pre-strain will be studied on the light absorptivity of the corrugated P3HT-based thin films under tensile strains. Last, the DC-based strain sensing capability of the flexible sensor will be validated by subjecting the sensor to tensile loading and unloading cycles while measuring DC voltage generated from the sensor under broadband light.

## 2. Experiment Details

### 2.1. Materials

P3HT (regioregularity = 93–95%; Mw = 50–70 kDa) and amorphous (6,6)-phenyl-C61-butyric acid methyl ester (PCBM) were purchased from Solaris Chem. (Quebec, Canada). PEDOT:PSS (product number: PH1000) was acquired from Heraeus Inc. (Hanau, Germany). Polydimethylsiloxane (PDMS; product number: Sylgard 184 kit) was obtained from Dow Corning (Midland, MI, USA). Fluoro surfactant (product number: FS-30) was purchased from Chemours (Wilmington, DE, USA). Gallium-indium eutectic (EGaIn), 1,2-dichlorobenzene (DCB), Dimethyl sulfoxide (DMSO) were purchased from Sigma-Aldrich (Darmstadt, Germany). Other chemicals used in this study were obtained from Fisher Scientific (Hampton, NH, USA).

### 2.2. Preparation of Test Specimens

#### 2.2.1. Corrugated PEDOT:PSS Conductive Thin Films

A total of 23 corrugated PEDOT:PSS thin films were prepared by varying pre-strain and the number of PEDOT:PSS thin film layers. Each test specimen is named as shown in Table 1. On one hand, six different pre-strains (i.e., 1%, 3%, 5%, 10%, 15%, and 20%) were used to fabricate the corrugated thin film to exhibit six different thin film buckling characteristics. On the other hand, five different thicknesses of PEDOT:PSS thin film were prepared by varying a deposited number of PEDOT:PSS layers from 1 to 9 layers with a 2-layer interval. For each test case, one corrugated PEDOT:PSS conductive thin film specimen was prepared.

The fabrication procedure began from the preparation of PEDOT:PSS conductive dispersion. 100 mL of conductive PEDOT:PSS dispersion was prepared by blending 95 mL of PH1000, 5 mL of DMSO, and 11 drops of fluoro surfactant, which was followed by manual agitation. The prepared conductive PEDOT:PSS dispersion was filtered using a 0.45 μm polyvinylidene fluoride (PVDF) filter to remove agglomerations that are larger than the pore size (0.45 μm) of the PVDF filter membrane.

PDMS substrates were prepared on 100 mm diameter silicon wafers. First, silicon wafers were cleaned using isopropyl alcohol (IPA) and dried with compressed air. Second, a PDMS mixture was prepared by blending 10 g of PDMS silicon base (part I) and 1 g of PDMS curing agent (part II) and manually stirring it with a glass rod for 2 min. The PDMS mixture was degassed under vacuum in a desiccator for 1 h. Third, the degassed PDMS was poured over the clean silicon wafer with extra caution not to entrap any air bubbles in the PDMS casted on silicon wafer. The PDMS casted silicon wafer was transferred to the vacuum oven to anneal at 80 °C for 2 h to crosslink the PDMS. The disk-shape PDMS substrate (diameter = 100 mm and thickness = 1 mm) was carefully removed from the silicon wafer. Then, rectangular PDMS substrates (length = 75 mm and width = 12.5 mm) were cut from the disk-shape PDMS substrate.

The rectangular PDMS substrate was pre-strained by manually stretching the PDMS substrate to the desired magnitude of tensile strain and fixed onto a glass slide (length = 75 mm and width = 25 mm) with two paper clips. Air bubbles at the interface between the PDMS substrate and the glass slide were carefully removed by rubbing the PDMS surface with nitrile-gloved fingers. The pre-strained PDMS substrate was cleaned again with IPA and then dried with compressed air. Lastly, the pre-strained PDMS substrate was treated with UV ozone cleaner for 2 h to improve wettability of the PDMS substrate.

The pre-strained PEDOT:PSS thin film on the glass slide was mounted in the spin coater immediately after completion of the UV ozone cleaning process. The conductive PEDOT:PSS dispersion was dispensed over the entire area of the pre-strained PDMS substrate after filtering using a 0.45 μm pore-size PVDF filter membrane. The thin film deposition was performed using the spin-coating process, which consists of two steps to spread solution on the substrate and spin the solution on the substrate to coat thin films. The first step began by spreading the PEDOT:PSS dispersion at 300 revolutions per minute (rpm) for 50 s, during which spin speed was linearly increased from 0 to 300 rpm for 10 s and maintained at 300 rpm for 40 s. Consequently, the second step began by spinning the pre-strained PDMS covered with PEDOT:PSS dispersion at 750 rpm for 30 s. During the spinning process, moisture was being dried out from the PEDOT:PSS dispersion. The spinning process continued by increasing the spin speed from 750 rpm to 1000 rpm for 15 s and maintaining the spin speed to be 1000 rpm for 2 min. Finally, the deposited PEDOT:PSS thin film was annealed in a vacuum oven at 100 °C for 10 min. This process completed an one layer deposition of the conductive PEDOT:PSS thin film. Specimens having more than one layer were fabricated by repeating the process for the subsequent PEDOT:PSS layer after cooling the thin film for 2.5 min.

#### 2.2.2. Corrugated P3HT:PCBM Photoactive Thin Films

To fabricate corrugated P3HT:PCBM photoactive thin film, P3HT:PCBM solution was prepared. 1:1 w/v % P3HT:PCBM solution was prepared by dissolving P3HT and PCBM in DCB. First, 3 w/v % P3HT and 3 w/v % PCBM solutions were prepared by dissolving 0.15 g of P3HT and 0.15 g of PCBM in each of 5 mL DCB, respectively. The solutions were heated at 45 °C and stirred at 450 rpm for 72 h. To minimize degradation of the solutions, vials containing the solutions were wrapped with paraffin film and aluminum foil. Then, the solutions were cooled down to room temperature and filtered using 0.45 μm polytetrafluoroethylene (PTFE) filter membrane to remove possible agglomerations. To produce 1:1 w/v % P3HT:PCBM photoactive solution, the 3 w/v % P3HT solution, 3 w/v % PCBM solution, and DCB were blended by 1:1:1 by volume and manually agitated.

The corrugated P3HT:PCBM photoactive thin films were fabricated on pre-strained PDMS substrates (length = 75 mm and width = 12.5 mm). A total of six P3HT:PCBM thin film specimens were prepared with a single layer at six different pre-strain levels of 1%, 3%, 5%, 10%, 15%, and 20% (Table 2). The prepared 1:1 w/v % P3HT:PCBM solution was dispensed, after filtering using a 0.45 μm PTFE membrane, uniformly over the entire area of the pre-strained PDMS substrate. Deposition of P3HT:PCBM photoactive thin films was performed using a similar spin-coating process used for the deposition of PEDOT:PSS thin films. The only difference is a shorter spinning duration for 1 min, instead of 2 min, in the last step at the 1000 rpm spin speed. The deposited P3HT:PCBM thin films were annealed at 110 °C for 1 h in a vacuum oven. It should be noted that the P3HT:PCBM thin film was covered with a petri dish to retard the evaporation of DCB during the annealing process, which could enhance the optoelectronic properties of the P3HT:PCBM thin film.

#### 2.2.3. Flexible Thin Film Strain Sensor

**Figure 1:**
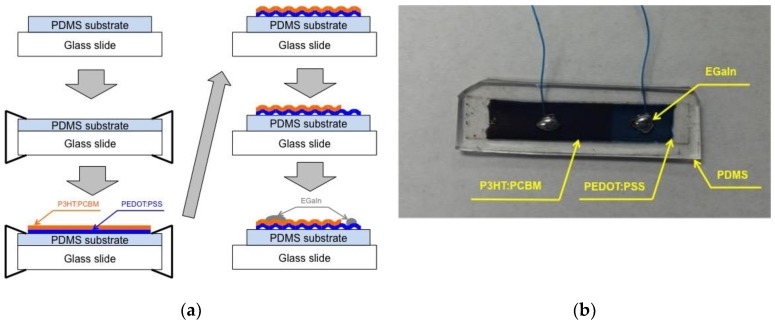
(**a**) A flexible strain sensor is fabricated by depositing corrugated P3HT:PCBM and PEDOT:PSS thin films on a pre-strained PDMS substrate. (**b**) The fabricated flexible strain sensor is shown from top view.

A total of six flexible strain sensors were fabricated by varying the number of PEDOT:PSS layers and pre-strain applied to the PDMS substrates (length = 75 mm and width = 12.5 mm) (Table 3). The sensor fabrication procedure (Figure 1a) began from the deposition of PEDOT:PSS conductive thin films onto the pre-strained PDMS substrate by following the same methodology described in Section 2.2.1. Once the desired number of PEDOT:PSS layers (i.e., 7 or 9 layers) were deposited onto the pre-strained PEDOT:PSS layers, a single layer of P3HT:PCBM photoactive thin films was coated onto the PEDOT:PSS layers by following same spin-coating procedure presented in Section 2.2.2. Then, one end of the coated P3HT:PCBM thin films was cleaned with DCB to expose a rectangular area (length = 20 mm and width = 12.5 mm). Then, the pre-strained PDMS substrate was released after removing the paper clips to produce the corrugated PEDOT:PSS and P3HT:PCBM thin films. One electrical connection was established on the conductive PEDOT:PSS thin films using EGaIn and 30 American wire gauge (AWG) wire. Another electrical connection was made on the P3HT:PCBM thin films using EGaIn and the 30 AWG wire. Finally, fabricated is a flexible strain sensor consisting of corrugated P3HT:PCBM and PEDOT:PSS thin films and two electrical connections.

The completed flexible strain sensor with the wires was encased in PDMS to minimize environmental effect and hold the electrical wire connections in place. To encase the flexible strain sensor with PDMS, the sensor was placed on a 100 mm diameter petri dish. Wires of the sensors were attached to the edge of the petri dish using a glue gun not to misplace the wires on the thin films during PDMS casting. Then, 22 g of PDMS mixture was prepared and carefully poured into the petri dish not to entrap air bubbles in the PDMS. The PDMS encasing was cross-linked by annealing in a vacuum oven at 80 °C for 2 h. Finally, the flexible strain sensor specimens were prepared after carving out the specimens from the PDMS encased sensors (Figure 1b).

### 2.3. Test Setup

#### 2.3.1. Characterization of Optical Transmittance of PEDOT:PSS at Tensile Strains

**Figure 2:**
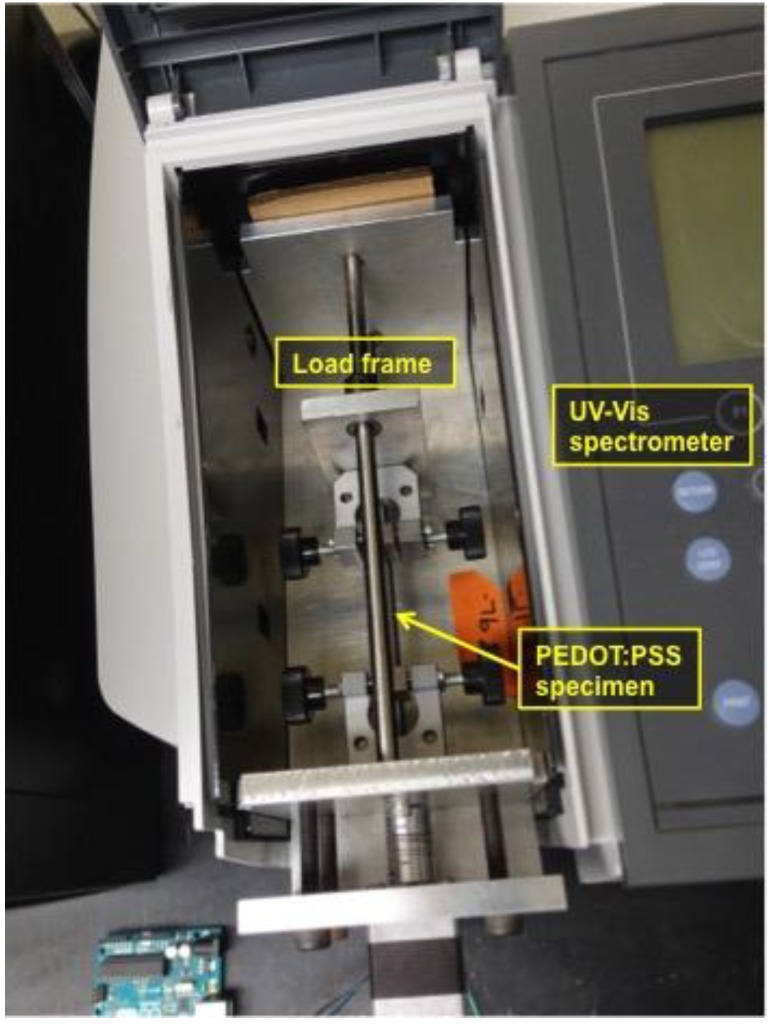
An UV-Vis spectrophotometer equipped with a custom-built load frame, which fits in measurement chamber, measures optical spectrum of the thin film specimens at various tensile strains.

The corrugated PEDOT:PSS thin films were interrogated with an ultraviolet-visible (UV-Vis) spectrophotometer (product name: UV-1800; Shimadzu, Kyoto, Japan) to acquire optical transmittance spectrums in the wavelength range from 300 to 900 nm. Eleven different UV-Vis transmittance spectrums were acquired at eleven different tensile strain levels during loading from 0% to a maximum strain, which is identical to a pre-strain level applied for fabrication of the corrugated PEDOT:PSS thin film specimen, and unloading to 0% using a custom-built load frame (Figure 2). The specimen was stretched and released by five equal steps during loading and unloading. As a result, a total of eleven UV-Vis transmittance spectrums were produced from one specimen.

#### 2.3.2. Characterization of Sheet Resistance of PEDOT:PSS at Tensile Strains

**Figure 3:**
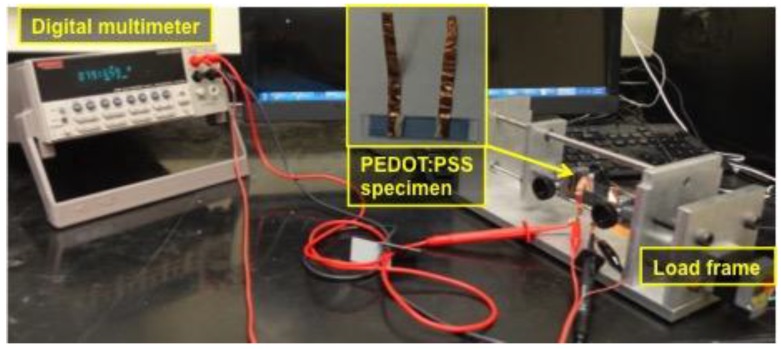
Electrical connections were established on the corrugated PEDOT:PSS thin film specimen to measure four-point probed resistance using a digital multimeter. The specimen is strained using the custom-built load frame to measure the resistance at various tensile strains.

To measure sheet resistance of the corrugated PEDOT:PSS thin film, two electrodes were established on the thin film using copper tape and silver paste as shown in the inset of Figure 3. Two straps of copper tapes wrapped the both ends of the PEDOT:PSS thin film specimen to make a distance between the copper tape electrodes 26 mm. Then, silver paste was applied at the margin of copper tape to ensure best electrical connections between copper tape and PEDOT:PSS thin film. After placing the silver paste at the edge of the copper tapes, the distance between two electrodes became 25 mm, which was used to calculate a sheet resistance at 0% strain using:

(1) Sheet ressitance=(Measured resistance × Width)/Length

Distance between the electrodes changed as tensile strain was applied onto the specimen while width of the specimen remained same. So, when the specimen was stretched, the length used for calculation of the sheet resistance was estimated—larger than 25 mm—by considering increase in the distance between two electrodes.

A digital multimeter (DMM; product number: Keithley 2700, Tektronix, Beaverton, OR, USA) was used to measure four-point probed resistance at various strains. Strain was applied using the custom-built load frame shown in Figure 3. Like optical transmittance characterization test, eleven strains were applied to the PEDOT:PSS thin film specimen during one cycle of loading and unloading. So, a total of 11 resistances were obtained and used for calculating sheet resistances.

Among 23 specimens, 15 specimens only with more than one layer were tested by stretching the specimen to desired tensile strain levels and measuring a sheet resistance at each strain level. Please note that two specimens (i.e., 15P5L and 20P3L) failed during testing. For the rest of 8 specimens with a single PEDOT:PSS layer, sheet resistances were measured but only at 0%.

#### 2.3.3. Characterization of Light Absorption of P3HT:PCBM at Tensile Strains

A test setup for characterization of UV-Vis light absorption of the P3HT:PCBM photoactive thin film specimen is identical to the test setup shown in Figure 2 used for optical transmittance characterization of the PEDOT:PSS thin film specimens. The measurement mode was light absorption, instead of transmittance, in the wavelength range from 300 nm to 900 nm.

#### 2.3.4. Validation of Strain Sensing of Flexible Strain Sensor

**Figure 4:**
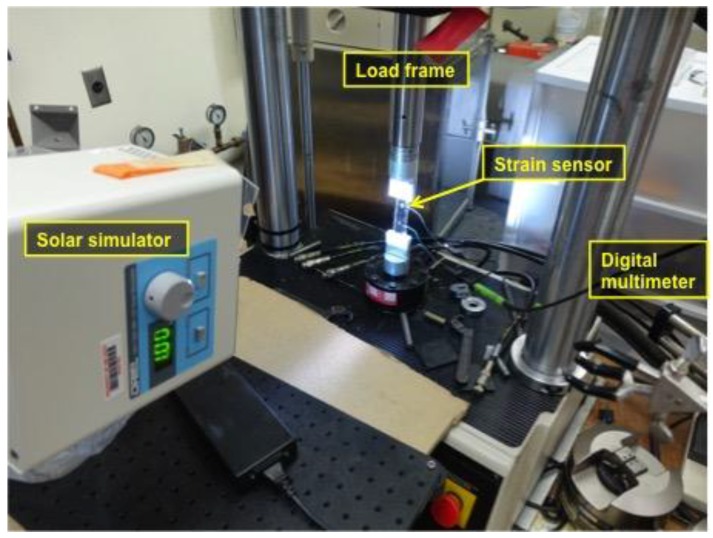
The flexible strain sensor was tested to validate its DC voltage-based strain sensing capability under tensile loading and unloading cycles while measuring DC voltage under one-sun light.

The fabricated flexible strain sensors were first subjected to quality test by measuring current-voltage (IV) responses using a source-measurement unit (SMU; product number: Keithley 2450, Tektronix, Beaverton, OR, USA) under one-sun light from a solar simulator (product name: Oriel 7320 LED solar simulator, Newport, Irvine, CA, USA) and without light. Then, qualified strain sensors were tested to validate strain sensing capability using the test setup shown in Figure 4. To apply cyclic tensile loading and unloading to the flexible strain sensor, a servo-hydraulic load frame was used. DC voltage was measured from the sensor using the digital multimeter under one-sun light from the solar simulator. The testing room was maintained as dark as possible to minimize effect by ambient light although the ambient light is thought to have minimal effect on the strain sensing of the sensor. It is because of the relatively weaker intensity of the ambient light than the one-sun light from the solar simulator.

## 3. Results and Discussion

### 3.1. Optical Transmittance of PEDOT:PSS at Tensile Strains

**Figure 5:**
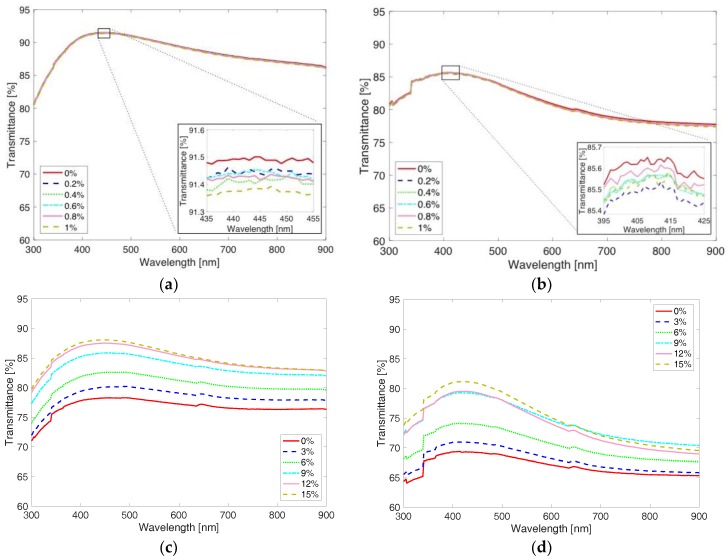
Light transmittance spectrums are shown at various tensile strains during loading. The spectrums are acquired from specimens (**a**) 1P1L, (**b**) 1P9L, (**c**) 15P1L, and (**d**) 15P5L.

Figure 5 shows four representative sets of light transmittance spectrums that were acquired at six different tensile strains during only the loading cycle to clearly present how the transmittance changes with the applied tensile strain. It should be noted that the light transmittance during unloading exhibited similar trends with the applied tensile strain. By comparing transmittance spectrums of the 1% pre-strain specimens shown in Figure 5a,b to the 15% pre-strain specimens shown in Figure 5c,d, one can understand that overall transmittance of the specimens fabricated at 1% is higher than the transmittance of the specimens prepared at 15% pre-strain. In addition, it can be seen that overall transmittance decreases as there are a greater number of layers. Also, the overall transmittance increases as the tensile strain applied to the specimen increases, which is shown more clearly in Figure 5c,d due to larger strain applied up to 15%.

**Figure 6:**
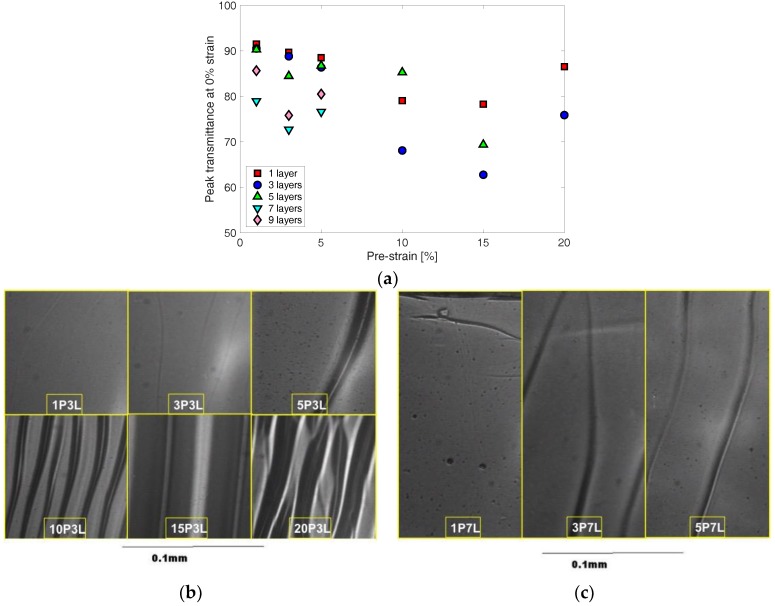
(**a**) Peak transmittances at 0% strain are shown with pre-strain that was applied when fabricating corrugated PEDOT:PSS thin film specimens. Optical microscopic images are shown for the corrugated thin film specimens having (**b**) 3 layers and (**c**) 7 layers.

In Figure 6a, the peak transmittance at 0% strain is shown with a pre-strain, at which the corrugated PEDOT:PSS thin film specimen was fabricated. The overall peak transmittance at 0% strain decreases as the pre-strain increases up to 15%. It increases again as the pre-strain increases from 15% to 20%. The specimens with 7 and 9 layers of PEDOT:PSS thin films exhibit different characteristics from the other specimens with a less number of layers (i.e., 1, 3, and 5 layers). For specimens with a large number of layers (i.e., 7 and 9 layers), peak transmittance decreases with the pre-strain increasing from 1% to 3% but increases again with the pre-strain increasing from 3% to 5% strain. The decreasing transmittance trend with increasing pre-strain from a specimen seems to be mainly due to wrinkles formed in the corrugated thin films.

Six specimens having three layers of PEDOT:PSS thin films, which were fabricated at six different pre-strains, were observed using an optical microscope (Figure 6b). Wrinkles are shown in all 6 specimens. But, for the three specimens fabricated at low pre-strain (i.e., 1%, 3%, and 5%), depth as well as density of wrinkles are smaller than ones in other specimens prepared at higher pre-strain (i.e., 10%, 15%, and 20%). Therefore, the peak transmittances at 0% strain of the higher pre-strain specimens are smaller than lower pre-strain specimens’ transmittance due to more populated and deeper wrinkles. This is because the higher pre-strain exceeded the critical buckling strain of the PEDOT:PSS thin film while the smaller pre-strain was not large enough to induce buckling. Up to 15% pre-strain, the peak transmittance shows a decreasing trend with increasing pre-strain because more and deeper wrinkles are formed as the pre-strain increases. However, peak transmittance increases again from 15% to 20% for both 1 layer and 3 layers specimens, which is even higher than the peak transmittance at 10% pre-strain. This seems to be due to shallower or less wrinkles in 20% specimens compared to 10% and 15% specimens. On the other hand, peak transmittance of specimens having a larger number of layers decreases when the pre-strain increases from 1% to 3% but increases again when pre-strain increases from 3% to 5%. While 3P7L and 5P7L specimens show a similar number of wrinkles in the limited viewfinder in Figure 6c, wrinkles in 5P7L seem to be a little shallower than the ones in 3P7L, which could result in higher peak transmittance at higher pre-strain.

**Figure 7:**
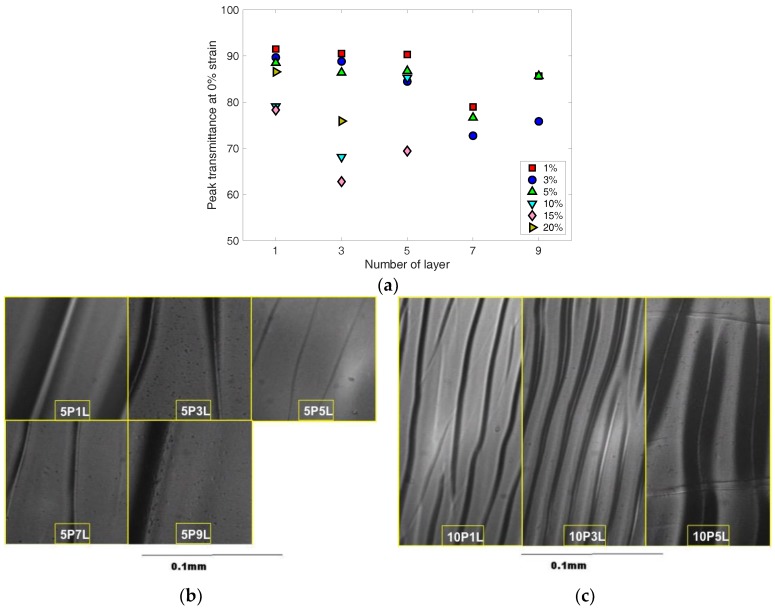
(**a**) Peak transmittances at 0% strain are shown with the number of PEDOT:PSS layers. Optical microscopic images are shown for the corrugated thin films that were fabricated at (**b**) 5% pre-strain and (**c**) 10% pre-strain.

The peak transmittance data are plotted with respect to the number of layers in Figure 7a. All three sets of corrugated PEDOT:PSS thin film specimens, which were fabricated at low pre-strain (i.e., 1%, 3%, and 5%), exhibit a similar trend in change of peak transmittance with increasing number of layers. Up to 5 layers, the change in peak transmittance was small. However, an abrupt decrease by ~10% in peak transmittance was observed as the number of layers increase from 5 to 7. Then, the peak transmittance increases again with the number of layer increasing from 7 to 9. It was shown that, for non-corrugated PEDOT:PSS thin films, peak transmittance decreased with an increasing number of layers (i.e., increasing thickness of thin film), which could be explained by the Beer–Lambert law as previously reported in [22]. However, the corrugated PEDOT:PSS thin films show a non-linear trend, which can be due to the wrinkles’ optical interference in the corrugated thin films.

To qualitatively investigate the wrinkles’ effect on the optical transmittance, optical microscopic images of 5% pre-strain specimens were taken and shown in Figure 7b. One can see that the wrinkles’ depth decreases as the number of layers increases up to 5 layers. The consistent light transmittance observed in the specimens with 5 or less number of layers seems to result from a cancelling effect between the decreasing wrinkle profiles and the increasing thickness of the thin films as the number of layers increases. Thicker thin films (i.e., thin films with larger number of layer) have higher critical buckling strain, which results in the formation of lower wrinkle profiles at the same pre-strain than thinner thin film. The abrupt decrease in transmittance from 5 to 7 layers seems to be due to increased wrinkle profiles in addition to increasing thickness of the thin film. Increased peak transmittance from 7 to 9 layers could result from the lower wrinkle profiles formed in the thin films due to higher critical buckling strain. On the other hand, high pre-strain specimens show different trend in peak transmittance change with the number of layers. The representative 10% pre-strain specimens’ optical microscopic images are shown in Figure 7c. Comparing 1 layer and 3 layers specimens, there are more wrinkles with deeper profiles in 3 layers specimen, which results in a decrease of peak transmittance. When the number of layers increased from 3 to 5, the peak transmittance increased again due to decreased density of wrinkles.

**Figure 8:**
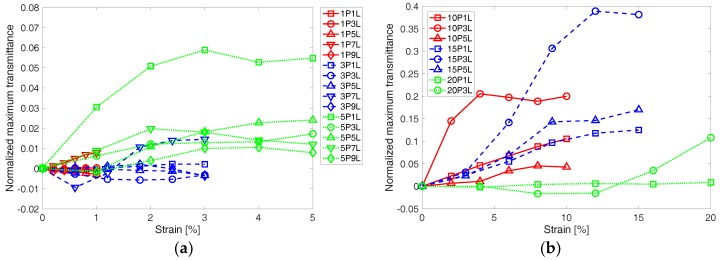
Normalized maximum transmittance change is shown with applied strain for the corrugated PEDOT:PSS thin film specimens, which were fabricated at (**a**) low pre-strain up to 5% and (**b**) high pre-strain up to 20%.

In addition, to understand the effect of tensile strain applied to the corrugated PEDOT:PSS thin film on light transmittance of the thin film, the peak transmittance acquired at each of the applied tensile strains was normalized using:

(2) $$T_{NP}=\left(T_{P}-T_{P,0\%}\right)/T_{P,0\%}$$

where, *T_NP_* is normalized peak transmittance, *T_P_* is peak transmittance at a strain, *T_P_*_,0%_ is peak transmittance at 0% strain. The normalized peak transmittance is shown with the applied tensile strain in Figure 8. Assuming that the corrugated PEDOT:PSS thin film becomes more flat as higher strain is applied, one can think that light transmittance increases due to less optical interference by the wrinkles. This trend is commonly observed in Figure 8a,b for small and large pre-strain cases, respectively. Some specimens exhibit a much larger increase or even a decreasing trend with increasing strain. These could be due to cracks, which were formed locally in the UV-Vis interrogation beam path, or too deep wrinkles that did not flatten enough to affect the transmittance during the loading.

### 3.2. Sheet Resistance of PEDOT:PSS at Tensile Strains

**Figure 9:**
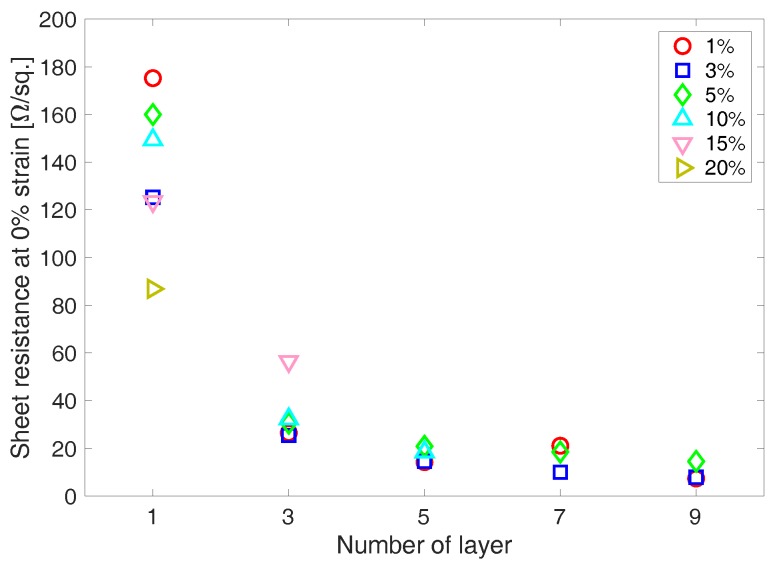
Sheet resistances of the corrugated PEDOT:PSS thin film specimens exponentially decreases as the number of deposited PEDOT:PSS layer increases. Pre-strain shows the strongest effect in sheet resistance for the 1 layer case. But, it becomes weaker as the number of layers increase.

Sheet resistance of the corrugated PEDOT:PSS thin film shows a strong relationship with the number of PEDOT:PSS layers (Figure 9). Similar to the sheet resistance of the inkjet-printed PEDOT:PSS thin films reported in [22], sheet resistance of the corrugated PEDOT:PSS thin films exhibited an exponential decrease with the increasing number of layers. In particular, the corrugated PEDOT:PSS thin films with 5 or more layers showed sheet resistance below ~20 Ω/sq. In particular, there was negligible effect by the pre-strain for the thin films with 5 or more layers while the specimens with less than 5 layers were affected by the pre-strain. It is because the wrinkle profiles of the corrugated PEDOT:PSS thin film specimens with less than 5 layers change more drastically with the pre-strain due to the smaller critical buckling strain than the PEDOT:PSS thin film specimens with 5 or more layers. Sheet resistance of the corrugated PEDOT:PSS thin film specimens with 1 layer exhibit the largest decrease with increasing pre-strain. This is due to a larger discrepancy between lengths based on the direct distance and the actual, which is contoured along the wrinkled thin films, length between the electrodes of the PEDOT:PSS thin film specimens as pre-strain increases. It should be noted that the corrugated thin film’s actual length (i.e., contoured) is longer than the length directly measured between two points (i.e., two electrodes). Sheet resistance was calculated with the shortest length between electrodes, instead of the contoured length. The higher pre-strain thin films tend to have more wrinkles and thus larger difference between the shortest length, which was used for the calculation of sheet resistance, and the contoured length. The length difference resulted in the decrease in sheet resistance of the 1 layer specimen with increasing pre-strain.

**Figure 10:**
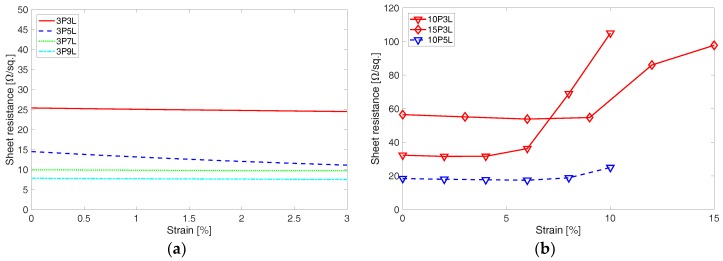
Effect of applied tensile strain on the sheet resistance is shown for (**a**) the 3% pre-strain specimen and (**b**) high pre-strain specimens fabricated at 10% and 15% pre-strain.

In Figure 10, results on change in sheet resistance of the corrugated PEDOT:PSS thin film specimens are shown with tensile strain applied to the specimens. Figure 10a shows representative results on how sheet resistance changes with applied tensile strain for small pre-strain specimens. The 3% pre-strain specimen’s sheet resistance is barely affected by the applied tensile strain. Large pre-strain specimens also show quite a consistent sheet resistance up to half of the applied maximum tensile strain (Figure 10b). But, as the applied tensile strain increases beyond 5%, sheet resistance increases, which seems to be due to the formation of crack openings. The crack onset at 5% or higher tensile strain could jeopardize sensing capability of a sensor due to the increasing sheet resistance. While the corrugated PEDOT:PSS thin film shows improved mechanical resiliency compared to non-corrugated PEDOT:PSS thin film, it could be improved furthermore through functionalization with nanomaterials [24,25].

### 3.3. Light Absorption of P3HT:PCBM at Tensile Strains

**Figure 11:**
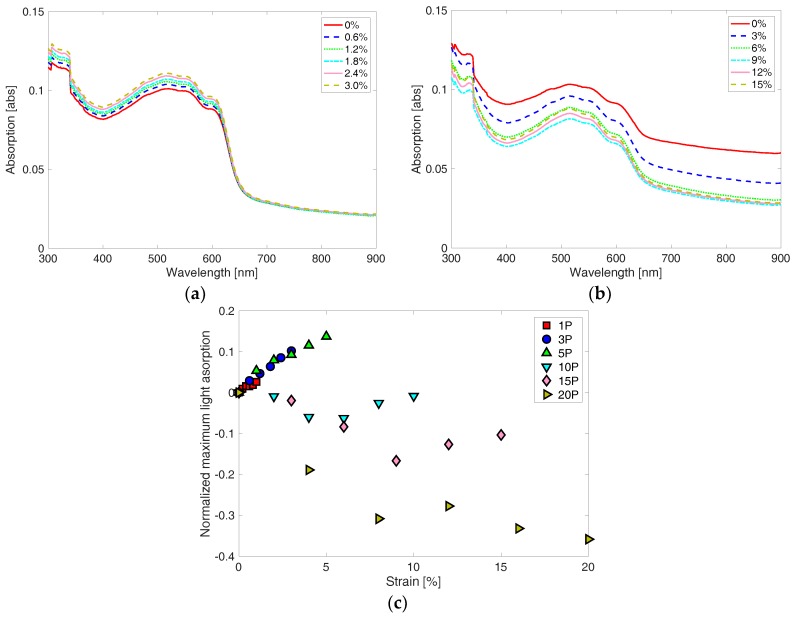
Light absorption spectrums of (**a**) 3P and (**b**) 15P P3HT:PCBM thin film specimens are shown at various strains from 0% to pre-strain levels, which are 3% and 15%, respectively. (**c**) Corrugated P3HT:PCBM thin film specimens that are fabricated at low pre-strain show increasing trend, while other specimens fabricated at higher pre-strain (i.e., above 10%) show decreasing trend, as the applied strain increases.

Light absorptivity of the corrugated P3HT:PCBM thin film shows different characteristics depending on magnitude of the pre-strain applied for the fabrication of the corrugated thin film. In Figure 11a,b, two representative UV-Vis light absorption spectrums are shown from 3% and 15% pre-strain specimens at various tensile strains during loading cycle. The 3% pre-strain specimen shows increasing light absorption with increasing tensile strain while the 15% pre-strain specimen shows decreasing light absorption with increasing tensile strain. The different characteristics of the corrugated P3HT:PCBM thin films are summarized in Figure 11c. The linear responses of the peak light absorption of the small pre-strain specimens are quantified by calculating strain sensitivities using:

(3) $$SS_{A}=\frac{\left(A_{P}-A_{P,0\%}\right)/A_{P,0\%}}{\varepsilon }$$

where, *SS_A_* is strain sensitivity of peak absorption, *A_P_* is peak absorption at a strain, *A_P_*_,0%_ is peak absorption at 0% strain, and *ε* is applied tensile strain. The light absorption-based strain sensitivities are presented in Table 4. The strain sensitivities are similar to the values reported from the non-corrugated P3HT:PCBM thin films [23]. One can understand that low pre-strain P3HT:PCBM thin film specimens did not form as many wrinkles as the high pre-strain P3HT:PCBM thin film specimens. However, high pre-strain P3HT:PCBM thin film specimens exhibit a dramatic decrease in light absorptivity with increasing applied tensile strain, up to half of the maximum applied strain, which seems mainly because wrinkles become more flat at a higher strain. The increasing trend in light absorption with the applied strain at higher than half of the maximum applied tensile strain is thought to result from the strained thin film after the wrinkles become completely flat. It was reported that P3HT molecules are ordered in more favorable micro-structure to result in the improved optical property of the P3HT-based thin film as the non-corrugated P3HT-based thin films are strained more [23]. The continuous and most significant decrease in light absorption of the 20% specimen seems to be attributed mainly to the formation of cracks in the thin films.

### 3.4. DC-Based Tensile Strain Sensing of Flexible Strain Sensor

**Figure 12:**
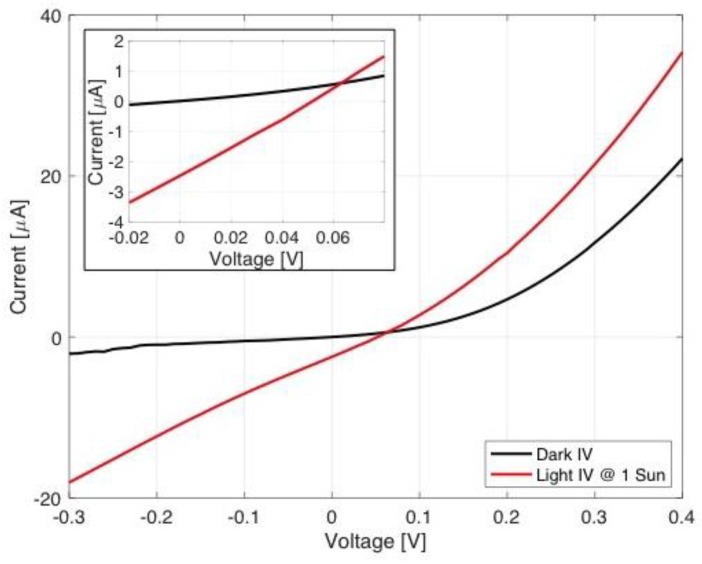
Current-voltage response of FS-1P7L specimen is shown under one-sun light and without light. The inset provides a clear view of the short circuit current and open circuit voltage from the vertical axis intercept and the horizontal axis intercept.

The corrugated PEDOT:PSS thin film specimens, which were fabricated with 7 or more layers, exhibited the lowest sheet resistance and maintained the sheet resistance to be low regardless of the amount of pre-strain. On the other hand, the corrugated P3HT:PCBM thin film specimens fabricated at 1%, 3%, or 5% pre-strain exhibited light absorption increasing proportionally with increasing applied tensile strain. Based on the information, only six different flexible strain sensors were fabricated at 1%, 3%, or 5% pre-strain with 7 or 9 layers of corrugated PEDOT:PSS thin film. To validate the performance of radiant-electric energy conversion performance of the fabricated flexible strain sensor, IV responses were obtained. In Figure 12, dark and light IV responses are shown for the flexible strain sensor, which was fabricated using a single layer of corrugated P3HT:PCBM thin film and 7 layers of the corrugated PEDOT:PSS thin films at 1% pre-strain. The inset of Figure 12 presents an expanded view of the IV responses in the range where short circuit current (i.e., absolute value of y-intercept; ~2.5 μA) and open circuit voltage (i.e., x-intercept; ~0.05 V) are clearly seen.

**Figure 13:**
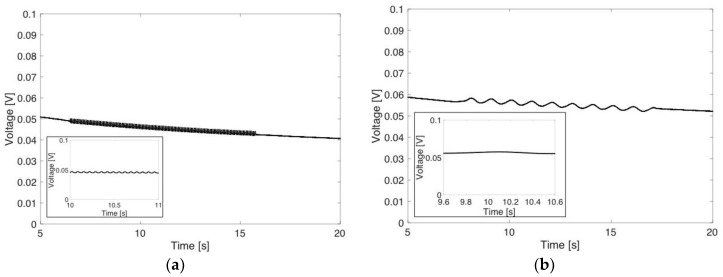
DC voltage responds to tensile loading and unloading cycles applied to FS-1P7L from 0% to 1% at (**a**) 1 Hz and (**b**) 15 Hz. Inset plots show the DC voltage response for 1 s time duration.

Figure 13 shows DC voltage response generated from the FS-1P7L flexible strain sensor in time domain when the strain sensor was subjected to sinusoidal tensile strain loading and unloading cycles. As the loading frequency increases from 1 Hz to 15 Hz while maintaining minimum and maximum tensile strains of 0% and 1%, respectively, cycles of the generated DC voltage also shift in frequency from 1 Hz to 15 Hz. DC voltage generated from the 1% pre-strain sensors (i.e., FS-1P7L and FS-1P9L) responds very well to the applied sinusoidal tensile strain loading and unloading cycles. The other four sensors, which were fabricated at higher pre-strains, produced a DC voltage sensor signal with lower signal-to-noise ratio than the 1% pre-strain sensors. It is thought to be mainly due to a disturbance in liquid EGaIn electrodes due to the higher strain applied.

**Figure 14:**
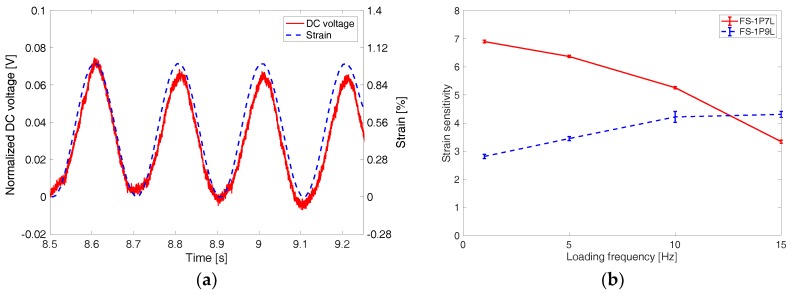
(**a**) DC voltage generated from FS 1P7L sensor varies with applied sinusoidal tensile loading and unloading cycles. (**b**) Strain sensitivities of the flexible sensor specimens are shown with loading frequencies.

In Figure 14a, the normalized DC voltage of FS-1P7L is presented with the applied load pattern at 5 Hz. Although there is slightly decreasing trend in DC voltage signal, it is clearly seen that overall DC voltage sensor responds well to the applied load pattern. This DC voltage-based strain sensing capability of the FS-1P7L sensor maintained even after two months from the fabrication. It is thought that PDMS encasement helped minimize the invasion of moisture and oxygen to the PEDOT:PSS and P3HT:PCBM thin films. Figure 14b shows how strain sensitivity (i.e., gauge factor) of the 1% pre-strain flexible sensors changes with the loading frequency. Although it is thought that the DC voltage-based strain sensing capability results from the P3HT:PCBM thin film’s light absorption varying with strain, strain sensitivities of the 1% pre-strain specimens are higher than the light absorption-based strain sensitivities of the P3HT:PCBM thin films, which are presented in Table 4. It seems that the higher DC voltage-based strain sensitivity is due to the increasing light transmittance of the corrugated PEDOT:PSS thin films with increasing tensile strain applied. It should be noted that the increased light transmittance of the corrugated PEDOT:PSS thin films at higher strain can allow more photons to pass through the PEDOT:PSS thin films to be absorbed by the P3HT:PCBM thin films, which consequently increased the DC voltage-based strain sensitivity of the sensor.

The varying strain sensitivity with loading frequency in Figure 14b could be mainly attributed to liquid EGaIn’s fluidic status, which can possibly lead to changes in the configuration of electrical connections due to applied loadings. Another possible reason for this varying sensitivity can be mechanical hysteresis of the corrugated thin films at a higher loading frequency. To further the analysis on the observed phenomena, more studies will be conducted in the future. In addition, it should be noted that the loading frequency was limited to 15 Hz due to limit in the capability of the load frame rather than the limit in sensing range of the devised flexible strain sensor. The upper limit in the loading frequency of the strain sensor was not checked in this study. 

## 4. Conclusions

In this study, a flexible strain sensor is designed using multilayered corrugated thin films using P3HT and PEDOT:PSS. The flexible strain sensor generated DC voltage under one-sun light from the solar simulator, and the DC voltage magnitude was shown to vary with the applied tensile strain up to 5% tensile strain. The flexible strain sensor was fabricated on the pre-strained PDMS substrate, where the multilayered thin films are deposited, to intentionally induce buckling in the deposited thin films after releasing the applied pre-strain for fabrication of the corrugated thin films. As a result, the best performance of the flexible strain sensor was achieved at 1% pre-strain with the highest gauge factor of 7 and the highest signal-to-noise ratio among the fabricated flexible strain sensors. Other flexible sensors fabricated at 3% and 5% also exhibited DC voltage-based strain sensing capability but with low signal-to-noise ratio.

In addition, each of the functional corrugated thin film constituents of the flexible strain sensor was characterized by measuring optical and electrical properties. First, the corrugated PEDOT:PSS thin films’ light transmittance and sheet resistance were studied with various fabrication design parameters (i.e., pre-strain and the number of PEDOT:PSS layer). It was shown that the corrugated PEDOT:PSS thin film’s optical transmittance is affected by both fabrication design parameters. Higher transmittance was acquired at lower pre-strain due to less populated wrinkles with shallower depths. As the number of layer (i.e., thickness) increases, a decreasing trend was observed in light transmittance. Yet, the lowest sheet resistance was achieved at 7 or 9 layers of the PEDOT:PSS thin film. In addition, the sheet resistance of the lower pre-strain corrugated thin film specimens was less affected by the applied tensile strain than higher pre-strain specimens regardless of the number of layers. Second, the lower pre-strain corrugated P3HT:PCBM thin film specimens exhibited increasing peak light absorption trend with increasing applied tensile strain while higher pre-strain specimens showed opposite trend. This provided design guidelines for the corrugated PEDOT:PSS thin film constituent when fabricating a flexible strain sensor to use 7 or 9 layers of corrugated PEDOT:PSS thin films and 1 layer of corrugated P3HT:PCBM thin films at 5% or smaller pre-strain.

## Figures and Tables

**Table 1:** Corrugated PEDOT:PSS conductive thin film specimens are named based on the amount of applied pre-strain and the number of layers.

Number of Layers	Pre-Strain (%)					
	1	3	5	10	15	20
1	P1L1	P3L1	P5L1	P10L1	P15L1	P20L1
3	P1L3	P3L3	P5L3	P10L3	P15L3	P20L3
5	P1L5	P3L5	P5L5	P10L5	P15L5	
7	P1L7	P3L7	P5L7			
9	P1L9	P3L9	P5L9			

**Table 2:** Corrugated P3HT:PCBM photoactive thin film specimens are named based on the amount of applied pre-strain.

Number of Layers	Pre-Strain (%)					
	1	3	5	10	15	20
1	P1	P3	P5	P10	P15	P20

**Table 3:** Flexible strain sensors are named based on the amount of applied pre-strain and the number of PEDOT:PSS thin films.

Number of PEDOT:PSS Layers	Pre-Strain (%)		
	1	3	5
7	FS-P1L7	FS-P3L7	FS-P5L7
9	FS-P1L9	FS-P3L9	FS-P5L9

**Table 4:** Strain sensitivity of the maximum light absorption of the corrugated P3HT:PCBM thin film specimens during loading cycle

Specimen (Number of Layer)	P1 (1)	P3 (3)	P5 (5)
Strain sensitivity	2.61	3.41	2.74
